# High Frequency Amplitude Detector for GMI Magnetic Sensors

**DOI:** 10.3390/s141224502

**Published:** 2014-12-19

**Authors:** Aktham Asfour, Manel Zidi, Jean-Paul Yonnet

**Affiliations:** 1 University Grenoble-Alpes, G2Elab, F-38000 Grenoble, France; E-Mails: Manel.Zidi@g2elab.grenoble-inp.fr (M.Z.); Jean-Paul.Yonnet@g2elab.grenoble-inp.fr (J.-P.Y.); 2 CNRS, G2Elab, F-38000 Grenoble, France

**Keywords:** magnetic sensor, Giant Magneto-Impedance (GMI), high-frequency amplitude detector, amplitude demodulation

## Abstract

A new concept of a high-frequency amplitude detector and demodulator for Giant-Magneto-Impedance (GMI) sensors is presented. This concept combines a half wave rectifier, with outstanding capabilities and high speed, and a feedback approach that ensures the amplitude detection with easily adjustable gain. The developed detector is capable of measuring high-frequency and very low amplitude signals without the use of diode-based active rectifiers or analog multipliers. The performances of this detector are addressed throughout the paper. The full circuitry of the design is given, together with a comprehensive theoretical study of the concept and experimental validation. The detector has been used for the amplitude measurement of both single frequency and pulsed signals and for the demodulation of amplitude-modulated signals. It has also been successfully integrated in a GMI sensor prototype. Magnetic field and electrical current measurements in open- and closed-loop of this sensor have also been conducted.

## 1. Introduction

Magnetic sensors are widely used in nearly all areas of engineering and industry. They are indeed suitable for a large palette of applications ranging from high-density magnetic recording to biomedical applications, non-destructive testing, automotive, space, military, security and scientific research applications [1]. Nowadays, a variety of these sensors with different physical principles is available. These mainly include inductive, fluxgate, Hall Effect, magneto-optical and giant magneto-resistance (GMR) sensors, *etc.* [1,2].

The Giant Magneto-Impedance (GMI) sensor is a relatively new category of magnetic sensors which has attracted the interest of the scientific community since the discovery of the physical phenomenon in the mid-90s [3]. The expected high sensitivity and resolution, the small size of the sensing element as well as the wide measuring bandwidth and the low power consumption are the main advantages of this technology [1,2,4,5].

The sensitive element of these sensors is a ferromagnetic amorphous material (a wire for example). Such a wire has a high frequency impedance which varies dramatically when it is subjected to an external magnetic field [3]. Actually, when the GMI wire is supplied by a high frequency current with constant amplitude, a voltage can be measured across this wire. If an external magnetic field is applied, this voltage is changed. This change is directly and only related to the impedance modification of the wire, since the current is of constant amplitude. Simply stated, this impedance modification is related to the skin effect in the wire in an intermediate frequency range of few MHz to few 10 MHz or 100 MHz. It is actually the penetration depth of the high frequency current which changes through the modification of the permeability of the wire induced by applied magnetic field. In higher frequency ranges, the phenomenon can be interpreted in terms of ferromagnetic resonance [6].

This GMI effect is immediately investigated for the design of magnetic sensors. These sensors are based on the impedance measurement of the sensitive element. Their implementation and their electronic conditioning are then relatively simple, in principle. A basic configuration requires a sensitive element, a high frequency oscillator and a voltage measurement circuit. A main element of this measurement circuit is the amplitude detector (or demodulator). Indeed, the voltage across the sensitive element is amplitude-modulated (AM) by the external measured magnetic field. After an amplitude detection (or demodulation) and amplification, the sensor output is obtained. This output reflects the measured field.

There are basically several kinds of amplitude detectors that are usually employed for the GMI sensors. The first kind includes the peak or amplitude detectors which are generally realized by diodes [7–16]. A second frequent kind, more sophisticated, uses traditional synchronous detection with commercial lock-in amplifiers [17–21], analog switches [22,23] or analog multipliers [17,24–26]. Other new categories of detectors like the Root-Mean-Square (RMS)-to-DC converters and the digital quadrature demodulators using a Software Defined Radio (SDR) have also been implemented in our recent works [27,28].

Each of these demodulation techniques has advantages and limitations. The lock-in amplifiers are relatively high-cost and sizeable. This is why they are usually more suitable and dedicated for laboratory general-purpose setups and tests of a variety of sensors including the GMI. Moreover, most synchronous detectors and lock-in amplifiers use analog multipliers which generally introduce additional noise and nonlinearities in the demodulation chain.

High performance digital demodulators (SDR), which can be built using a single board, successfully address all the limitations of analog synchronous detection [28]. They are therefore more suitable for embedded sensors and high sensitivity applications. However, the relative complexity of implementation; which requires the use of microcontrollers or Digital Signal Processors (DSP), may limit their use to specific applications where the desired signal-to-noise ratio (SNR) could not be achieved by traditional analog electronic conditioning. This may be the case in high sensitivity GMI sensors where the ultimate SNR is still dominated by the noise of the analog electronics rather than by the intrinsic noise of the sensitive element.

Among the different amplitude detectors, the peak detector is so far the easiest to implement. It is compact and inexpensive. It is well-renowned and widely used in GMI sensors. However, it is limited by the nonlinearity of the diodes (when the input signal is smaller than the threshold) and their temperature dependence. This threshold limitation can be reduced by using active rectifiers in which the diode needs to be included into the feedback loop of an operational amplifier (op-amp). However, the intrinsic structure of these diode-based active rectifiers presents generally the disadvantage of low input impedance, especially if they are used in high-speed application [29]. On the other hand, the performance of such rectifiers depends strongly on the rectifier load impedance [29].

The RMS-to-DC converters can also address the problem of the diode threshold since they are able to measure signals with amplitudes as low as a few tens of mV with high accuracy [27,30]. They are also easy to implement and require few components. They fit very well for a number of GMI sensor applications. However, despite their merits, these converters have a relatively small input bandwidth (less than few MHz) and a very small intrinsic demodulating bandwidth (1 to 5 Hz only) [27,30].

In this paper, an original concept of amplitude detector and demodulator for GMI sensors is presented. This concept combines a precision half-wave active rectifier, without using diodes, and a feedback circuit that ensures the amplitude detection with adjustable gain. The underlying idea is to propose a new detector design that addresses the limitations of passive and active diode-based detectors and of the RMS-to-DC converters. Also, compared to the synchronous detection and the SDR demodulation, the new design is obviously justified by the simplicity of implementation. This advantage, which is shared by the diode-based detectors and RMS-to-DC converter, is then retained.

The general architecture of a GMI sensor using this new detector will be given. The principles, the full electronic circuitry, a comprehensive theoretical study as well as the performance of the detector will be addressed throughout the paper. Finally, the GMI sensor, which integrates the new developed detector, will be used for magnetic field and electrical current measurements in open- and closed-loop operations.

## 2. Design and Implementation

### 2.1. The General Architecture of the Developed GMI Sensor

**Figure 1.**
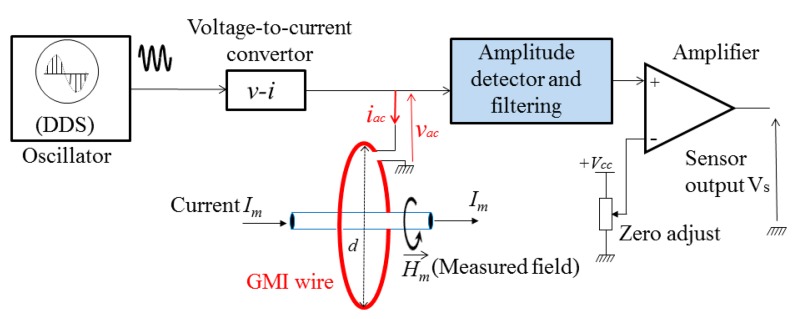
A block diagram of the GMI sensor including the new amplitude detector. The diameter of the core is *d* = 2 cm.

Figure 1 shows a block diagram of the developed sensor. The sensing element was a 100 μm diameter and 6.3 cm length ferromagnetic amorphous wire (Co-Fe-Si-B). This wire was curled to a core to form a loop of *d* = 2 cm diameter. The circumferential measured field *H⃗_m_* is produced by a current *I_m_* crossing the core.

The high frequency oscillator is based on a newly implemented technique for the GMI sensors [31]. This technique uses a Direct Digital Synthesizer (DDS) which allows generation of single frequency waveforms signals with high stability, high resolution and accuracy in frequency, amplitude, and phase.

The DDS voltage output is coupled to a new structure of voltage-to-current converter (*v-i*) which has been addressed in our recent work [32]. This voltage-to-current converter supplies the high frequency current, *i_ac_*, to the GMI element. The voltage, *v_ac_*, across the GMI wire is amplitude-modulated by the external measured field, *H*_m_. This voltage is applied to the input of the amplitude detector or demodulator. The demodulated voltage is then amplified by an instrumentation amplifier (AD620 from Analog Devices, Norwood, MA, USA) with zero adjust. The output of this amplifier, which reflects the measured field, can be set to zero for a zero measured magnetic field.

### 2.2. The Design and Performance of the High Frequency Amplitude Detector

**Figure 2.**
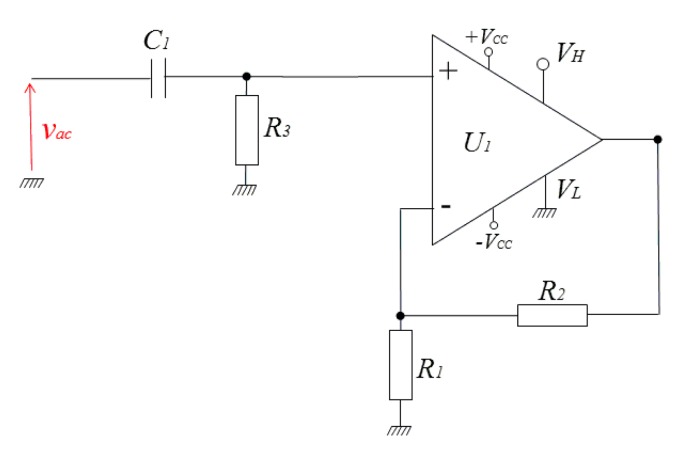
The electronic circuitry of the half wave rectifier. *U_1_*: OPA699, *C_1_* = 100 nF, *R_3_* = 50 Ω impedance matching), *R_1_* = 150 Ω, *R_2_* = 750 Ω.

The developed detector is based on the use of a wideband and high slew rate voltage-feedback op-amp (OPA699 from Texas Instruments, Dallas, TX, USA) that also has two output voltage limiters. Based on these voltage limiting capabilities, a half wave rectifier, with outstanding precision and speed, has firstly been implemented as shown in Figure 2.

The OPA699 op-amp has two limiting inputs (*V_H_* and *V_L_*). The output voltage of the op-amp is linearly dependent on the input when this output is between the limiter voltages *V_H_* and *V_L_*. In this case, the circuit behaves as a classical op-amp with an output dynamic range defined mainly by the power supplies ±*V_cc_* = ±5 V. When the output tries to increase beyond *V_H_* or *V_L_*, the corresponding limiter holds the output at *V_H_* or *V_L_*. The transition from the linear operation to the output limiting mode is very sharp and the recovery from the limiting mode or overdrive is very fast (1 ns). These features allow the op-amp to be used for both standard op-amp applications and nonlinear analog signal processing and high speed applications. A set of these applications includes the fast limiting Analog-to-Digital Converter input buffers, CCD pixel clock and video stripping, high frequency mixers and amplitude modulation (AM) signal generation, *etc.*

**Figure 3.**
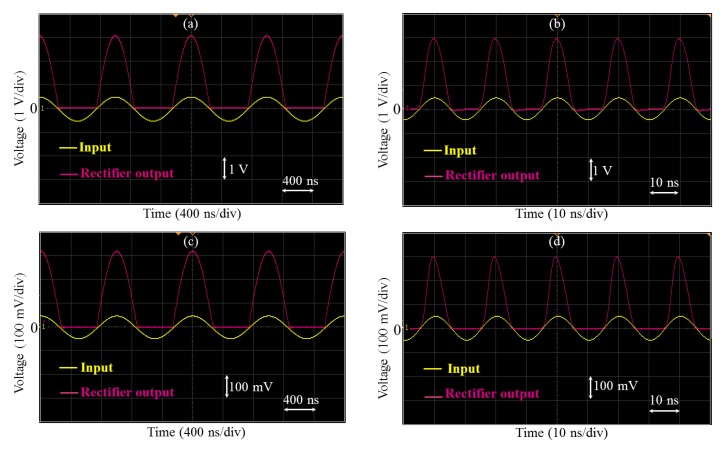
Half wave rectification and amplification of (**a**) 1 MHz/500 mV; (**b**) 50 MHz/500 mV; (**c**) 1 MHz/50 mV and (**d**) 50 MHz/50 mV sine waveforms The non-inverting gain was fixed to about 6. The signals were visualized on a digital oscilloscope.

In this current work, the op-amp is first used as an intermediate frequency (IF) limiting amplifier. For the implementation of a half wave rectifier (Figure 2), the positive limit is set to a default value of 3.5 V (*V_H_* pin is left open), while the negative limit is set to ground. In this way, only the positive half of the input voltage is retained at the output with the non-inverting gain. Figure 3a,b show an example of rectified signals of 1 MHz and 50 MHz when the input amplitude is about 500 mV. The rectified signals are also amplified by the gain of the non-inverting amplifier (a gain of 6 was used as an example).

In addition to its ability to perform simultaneous rectification and amplification of very high-frequency signals, this high-performance limiter allows also the rectification of very weak signals with amplitudes as low as some 10 mV (the limiter offset error is about ±10 mV). This is illustrated in Figures 3c and 3d where 1 MHz and 50 MHz signals of only 50 mV of amplitude were successfully rectified and amplified. This feature is a key advantage of the limiter when compared with diode-based rectifiers.

**Figure 4.**
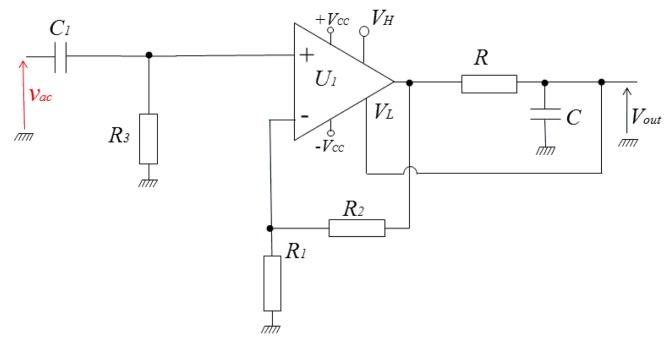
Amplitude detection of the rectified output. The mean value of the output, obtained by a low pass RC filter, is re-injected into the low-level limiter *V_L_*. *U_1_*: OPA699, *C_1_* = 100 nF, *R_3_* = 50 Ω impedance matching), *R_1_* = 150 Ω, *R_2_* = 750 Ω, *R* = 500 Ω, *C* = 100 nF.

The mean value of the rectified output can be easily obtained by a low-pass filtering. This mean value is obviously proportional to the input amplitude and hence to the measured field when the rectifier is used in a GMI sensor. However, the detection sensitivity, expressed in volt per unit of measured magnetic field, can be improved by exact amplitude detection rather than mean value measurement. A first solution for this amplitude detection of the rectified output is proposed in Figure 4. In this solution, the mean value of the rectified voltage is obtained by a low-pass RC filter. This mean value is re-injected into the low-level limiter *V_L_*.

**Figure 5.**
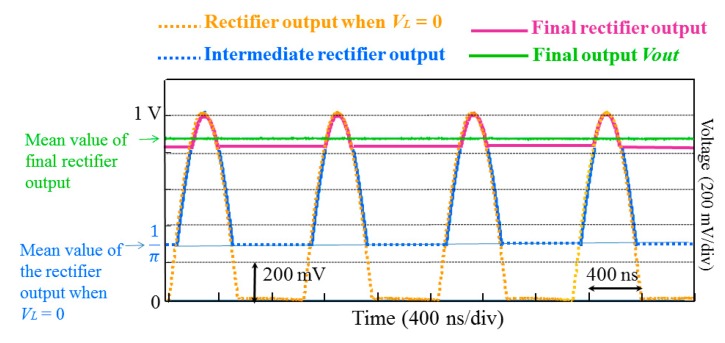
Operation principle of the rectifier with a feedback into the limiter *V_L_*. The brown curve was measured on an oscilloscope when *V_L_* = 0. The green curve was also measured in steady state when the filter output is re-injected to *V_L_* (circuitry of Figure 4). The blue and magenta curves were drawn and superimposed on the measured curves for illustration only.

Figure 5 illustrates this operation mode. At the initial time (*t* = 0), and when the power supply is firstly switched on, the voltage applied to *V_L_* is almost zero and the rectifier output follows the brown curve.

The amplitude of this voltage was intentionally fixed to 1 V to simplify the illustration. The mean value of this voltage is then 1/*π*. Starting from this initial time (*i.e.*, for *t*>0), this value is re-injected into *V_L_* and the rectified output will be now limited to a low level of 1/*π*. The output corresponds now to the blue intermediate curve in Figure 5, and its mean value is obviously higher than 1/*π*. During a short transient regime, the output will be progressively limited to higher and higher values, and its mean value will then be higher and higher. In steady state, the final output rectifier will approaches the peak of 1 V as illustrated by the magenta curve in Figure 5. The final mean value of the rectifier output is obtained at the output of the RC filter (green curve in Figure 5).

This first solution works well. The final mean value of the rectifier output approaches the amplitude of 1 V. However, some residual error between the output and the real peak of the voltage cannot be easily reduced. This is due to the fact that the rectifier gain is only fixed by the resistors *R_1_* and *R_2_*, and the obtained mean value will still naturally lower than the peak value. Adjusting the gain of the rectifier may reduce the relative error between the mean value and the peak value of the rectified output until some limit. The error of 20% was obtained for a gain of 6 and an error of 15% was obtained for very high gains (more than 40). Using high gains will reduce the bandwidth of the amplifier, which is not desirable. Moderate gain values should be used to take advantage of the high gain-bandwidth product of the limiting op-amp. Actually, while the solution proposed in Figure 4 gives reliably operation and measurements, it is however based on intuitive idea only. The use of the limiting amplifier in this unusual way (where its output is fed back to the limiting input *V_L_*) is not described by the manufacturer of the op-amp, neither the exact way how the limiting operation is performed. A rigorous comprehensive understanding requires more information about the internal structure of this limiter and how it controls the output, which is not provided by the manufacturer. It is therefore not possible to easily derive the equations that govern this operation principle and the final output.

For all these reasons, another original solution for the amplitude detection of the input was preferred and implemented. In this approach, the limiting op-amp is used according to the specifications and to the usual way of operation (the limiting input *V_L_* is set to a fixed and well-defined voltage). A theoretical comprehensive study is also possible which allows the prediction of the obtained results.

**Figure 6.**
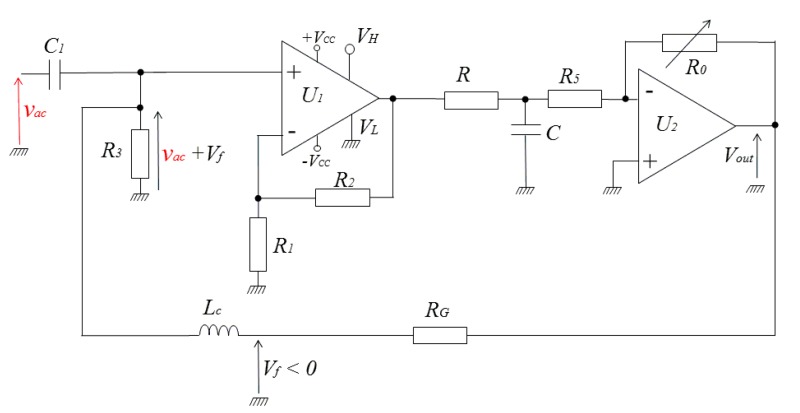
The electronic circuitry of the peak detector and optimized values at 1 MHz. *U_1_*: OPA699, *U_2_*: TL081, *C_1_* = 100 nF, *R_3_* = 1 k Ω, *R_1_* = 150 Ω, *R_2_* = 750 Ω *R* = 50 Ω *C* = 100 nF. *R_5_* = 200 Ω, *R_0_* variable 200 k Ω, *R_G_*: gain resistor. *L_c_* = 3 × 120 μH (choke inductor). The classical offset adjustment circuit of op-amp *U_2_* is also included (not shown in the figure).

Figure 6 shows the electronic circuitry of this second concept, where *v_ac_* is the sinusoidal input voltage of amplitude *V_ac_*. The values of the components are optimized for a working frequency of 1 MHz. In this circuitry, the mean value of the rectifier output is obtained by a 1st order low-pass filter, width moderate attenuation, formed by the resistor *R* and the capacitor *C*. The voltage at output of this filter is amplified and inverted by an inverting op-amp stage (*U_2_*). The gain of this stage is controlled by the resistors *R_5_* and *R_0_*. This stage behaves also as a second 1st order low-pass filter. In combination with the RC filter, it forms a 2nd order filter that improves the rejection of the high frequency carrier signal and its harmonics.

A negative feedback voltage *V_f_* (which reflects the output) is obtained from the output voltage, *V_out_*, through the voltage divider formed by *R_G_* and *R_3_*. This voltage is fedback to the input of the rectifier so as to form a closed-loop. The choke inductor, *L_c_*, guarantees the isolation between the high-frequency and DC sections. It acts as high impedance against the high frequency input *v_ac_*. This high frequency has not to reach the output of the detector via the branch of *R_G_*. The same choke inductor acts as a short circuit for DC currents. In other words, the DC feedback voltage, *V_f_*, must be supplied to the resistor *R_3_* without supplying AC voltage from *R_3_* to the detector output via the branch of *R_G_*. The choice of the value of the inductor depends on the frequency of *v_ac_*. In practice, and at a given frequency of the carrier, this choice is a trade-off between the value of the inductor (then its size) and the acceptable attenuation of the high frequency currents in the feedback circuit.

In this closed-loop, the rectifier ensures the function of comparator between the amplitude, *V_ac_*, of the input, *v_ac_*, and the *absolute value* of the negative feedback voltage *V_f_*. With appropriate adjustment of the loop parameters, the loop tends to cancel, or at least to minimize, the error ε between *V_ac_* and absolute value of *V_f_*. When the loop is well locked, this error is minimized. This means that the absolute value of *V_f_* is close to *V_ac_* and the final output voltage *V_out_* is locked to *V_ac_*. This negative output is then the image of the amplitude of the input. The gain between *V_out_* and *V_ac_* will mainly be defined by *R_G_* and *R_3_*.

**Figure 7.**
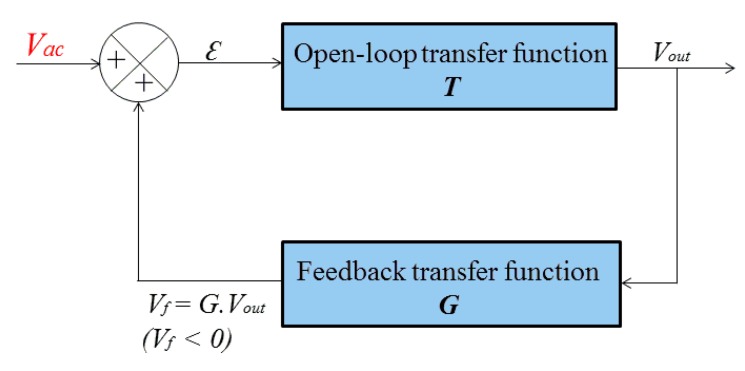
The functional diagram of the closed-loop of the amplitude detector.

A comprehensive study of this concept can be made using the functional diagram of the closed-loop given in Figure 7, where ***T*** and ***G*** denote the transfer functions of the open-loop and of the feedback, respectively. This is classical automatic control loop that measures the image of the output, via the measurement of *V_f_*, and compares this measurement to *V_ac_*. The loop is regulated to minimize the error ε (error in the sense of closed-loop operation between *V_ac_* and the absolute value of *V_f_*. This error can also be defined by the sum of *V_ac_* and *V_f_* since *V_f_* is negative as shown in Figure 7.

In this diagram, it is straightforward to express the output voltage by the Equation (1):

(1) $$V_{\text{out}}=T\cdot \varepsilon =T\cdot \left(V_{ac}+V_{f}\right)=T\cdot \left(V_{ac}+G\cdot V_{\text{out}}\right)$$

Rearranging terms yields a classical form of the transfer function of the system in closed-loop given by the Equation (2):

(2) $$\frac{V_{\text{out}}}{V_{ac}}=\frac{T}{1-G\cdot T}$$

The open-loop transfer function ***T*** is mainly defined by the gain of the rectifier, the low-pass filter response and the inverting amplifier gain. Based on the schematic of Figure 6, this transfer function is given by the Equation (3):

(3) $$T=(1+\frac{R_{2}}{R_{1}})\cdot \frac{1}{\pi }\cdot \frac{1}{\left(1+RC\cdot j\omega \right)}\cdot \left(\frac{-\frac{R_{0}}{R_{5}}}{1+\tau \cdot j\omega }\right)$$

where *ω* is the angular frequency of the amplitude *V_ac_* when it is time-dependent (*i.e*., in the case of amplitude-modulated input *v_ac_*), $j=\sqrt{-1}$, (1+*R*_2_/*R*_1_) is the gain of rectifier (gain of the non-inverting amplifier), 1/*π* is the factor relating the mean value of the half wave rectified voltage and its amplitude, $\frac{1}{\left(1+RC.j\omega \right)}$ and $\frac{-(R_{0}/R_{5})}{1+\tau \cdot j\omega }$ are the transfer functions of the low-pass RC filter and of the inverting amplifier, respectively, and *τ* is the time constant of the inverting amplifier transfer function (assuming a first order transfer function model).

For non-modulated input signals, *V_ac_* is time-independent and its angular frequency is null (*ω* = 0). In this case, the transfer function of the open-loop can be simplified according to Equation (4):

(4) $$T=T_{0}=\frac{V_{\text{out}}}{V_{ac}}=\left(1+\frac{R_{2}}{R_{1}}\right)\cdot \frac{1}{\pi }\cdot \left(-\frac{R_{0}}{R_{5}}\right)$$

On the other hand, the feedback transfer function ***G***, which is frequency independent in our design, is easily expressed by Equation (5):

(5) $$G=\frac{V_{f}}{V_{\text{out}}}=\frac{R_{3}}{R_{3}+R_{G}}$$

Equation (2) becomes:

(6) $$\frac{V_{\text{out}}}{V_{ac}}=\frac{T_{0}}{1-G\cdot T_{0}}\;\text{with}\;T_{0}<0$$

When the quantity |*G*·*T*_0_| in Equation (6) becomes very large with respect to one (|*G*·*T*_0_|≫1), the one can be neglected and Equation (6) reduces to Equation (7) which is the ideal relationship between the input and the output of the closed-loop system:

(7) $$\frac{V_{\text{out}}}{V_{ac}}\approx -\frac{1}{G}=-\frac{R_{3}+R_{G}}{R_{3}}=-\left(1+\frac{R_{G}}{R_{3}}\right)$$

Under the condition |*G*·*T*_0_|≫1, the output voltage, *V_out_*, depends linearly on the amplitude of the input voltage, *V_ac_*, with a proportionality constant (or gain) given by –(1+*R*_G_/*R*_3_). This closed-loop gain is only determined by the feedback circuit. It is a predictable and stable gain because the feedback circuit is implemented using only stable passive components rather than active components (active rectifier and inverting amplifier…). Since passive components have much better drift characteristics (temperature, power supply…) than active ones, the current concept of amplitude detection combines the advantages of classical active peak detectors (no diodes use and no threshold limitations) and the advantages of the low drift of passive components. This is one of the underlying ideas of this amplitude detector which allows hence an exact measurement of the input amplitude with an easily programmable gain.

**Figure 8.**
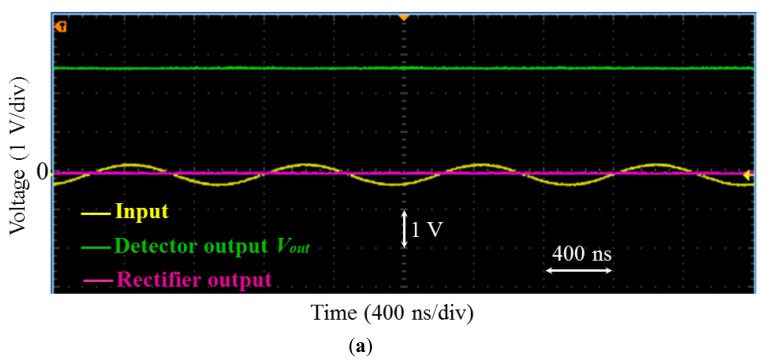

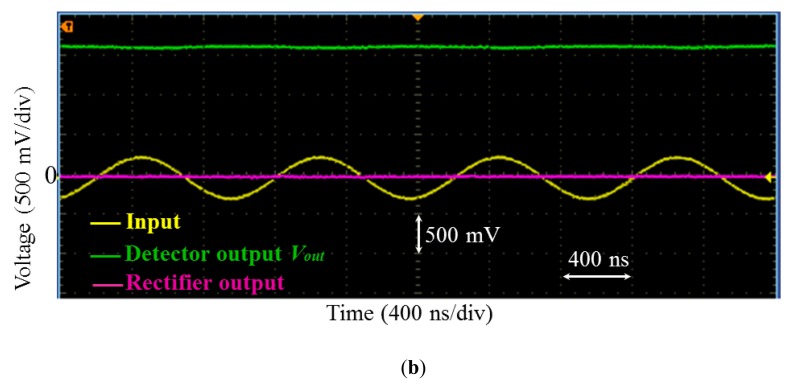
Input signal (yellow color), detector output (green color) and comparator output (magenta color) of a 1 MHz/250 mV signal for two values of feedback resistor. (**a**) *R_G_* = 10 kΩ; (**b**) *R_G_* = 5.6 kΩ.

These theoretically considerations have been experimentally validated. The transfer function, *T_0_*, of the open-loop was adjusted to about −950 through the adjustment of *R_0_*. The resistor *R_3_* was fixed to 1 kΩ Figure 8 shows the input sine waveforms and the obtained output of the detector for two different values of the resistor *R_G_* (*R_G_* = 10 kΩ and 5.6 kΩ).

**Figure 9.**
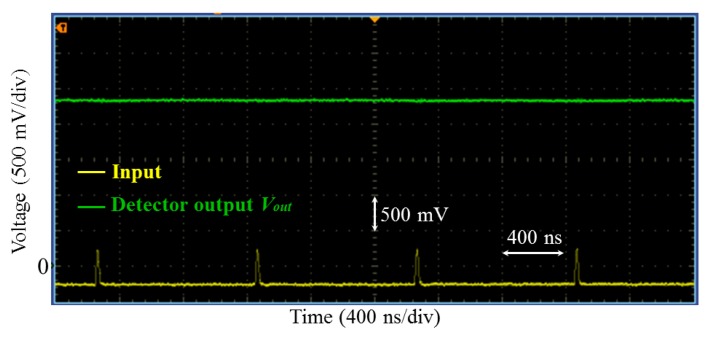
Peak detection of sharp pulses using the developed detector with *R_G_* = 10 kΩ. The pulse duration was less than 20 ns and the amplitude was about 225 mV. The repetition time of the pulse was of 1 μs.

The input signal was of 1 MHz of frequency and 250 mV of amplitude. Remember that voltage at the detector output is actually negative. However, for all the results addressed in the following sections and figures of this paper, this voltage has been intentionally inverted (green plots in Figures 8, 9 for example) for ease of explanation and clarity of visualization only. Only the absolutes values of this voltage and of the gains given by Equations (6) and (7) will be considered.

The measured outputs of the detector were approximatively about 2.6 V and 1.6 V for *R_G_* = 10 kΩ and 5.5 kΩ respectively. The closed-loop gains were then about 10.4 and 6.4, respectively. These values are close to the theoretical approximate predictions (11 and 6.6) given by Equation (7). The difference between the approximation in Equation (7) and the measurement is about 5.5% for *R_G_* = 10 kΩ and about 3% for *R_G_* = 5.6 kΩ. It is straightforward to verify that the error is larger for larger *R_G_*. Actually, the comparison between the measurement and the approximate formula was performed here using the same value of *T_0_* for both *R_G_* = 10 kΩ and *R_G_* = 5.6 kΩ. The product |*G*·*T*_0_| is then higher for *R_G_* = 5.6 kΩ. This leads to a smaller error between the measurement and the approximation of Equation (7). More generally, at a given feedback gain G, the error can be reduced by using high values for the open-loop gain *T_0_* (proportional corrector in the sense of automatic control loop). However, as it is well-known, in automatic control systems, increasing this gain is not always desired since it leads to instability problems of the closed-loop. To overcome this limitation, an integral corrector can be used. Combined with a proportional corrector, it perfectly cancels the error even with moderate proportional gains. In the current design, it is possible to include such integrator which realized by another op-amp circuit. This obviously adds more electronic components in the design. A trade-off between the error and the additional complexity has to be done.

The rectifier output (output of the op-amp OPA699) is also shown in Figure 8. This output reflects the error ε and we can see that it is very small since the loop is well locked. It may be worth noting that the detector output still exhibits small oscillations at 1 MHz. The origin of these oscillations is well identified. Actually, as mentioned above in this section, some of the high frequency currents coming from input are not totally blocked by the chock inductor *L_c_* in the branch of *R_G_*. Several standard solutions could be implemented to further minimize this effect. The first straightforward solution is to increase the value of the inductor. In another solution, based on the “pole insertion” technique, the isolation between the two sections can be improved by adding a capacitor in parallel to *L_c_*. If the resonance frequency of this parallel circuit is well-tuned to the working frequency, the resulting “trap” circuit exhibits very high impedance and becomes an efficient blocking circuit. However, the main limitation of this method may be the need to well-tune the resonance of the trap parallel circuit for each working frequency.

The developed circuit was also tested for peak detection of fast pulses. Actually, pulse excitation is a frequently used approach in GMI sensors as an alternative to single frequency excitation. This excitation mode allows several advantages. These considerations are out of the scope of the current paper, but the interested reader may refer to [8] and [33] for more details.

A train of sharp pulses (Figure 9) was applied to the input of the detector with *R_G_* = 10 kΩ. The pulse duration and its amplitude were about 20 ns and 225 mV, respectively. The repetition time of the pulse was of 1 μs. Figure 9 shows that the voltage at the detector output was about 2.33 V. This roughly yields a gain of about 10.4, which is close to the prediction given Equation (7).

**Figure 10.**
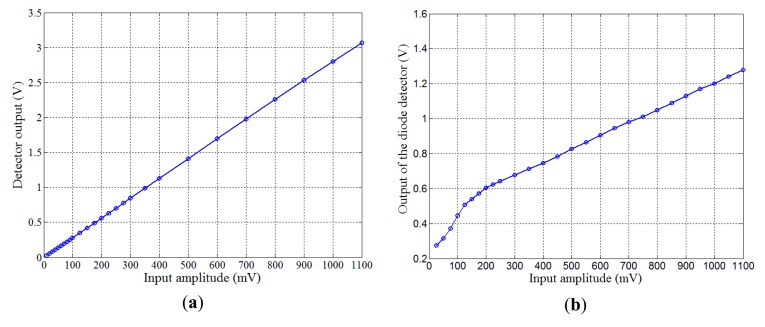
Comparison between the linearity of (**a**) the developed detector and (**b**) a commercial diode-based peak detector (LTC5507).

It is important to note that this detector ensures a very linear relationship between the amplitude of the input and the obtained output. In comparison with most of the commercially available diode-based peak detectors, the linearity of the detector is generally better, especially for low level amplitudes. Figure 10a shows input-output characteristics of the detector where the gain was fixed to about 3 only (*R_G_* = 2 kΩ) in order to allow for the study of large input range (without saturating the output). For comparison purposes, the measured input-output characteristic of a commercial and temperature-compensated peak detector (LTC5507 from Linear Technology) are plotted in Figure 10b. In addition to this good linearity, the detector is low-cost and compact. It also requires no reference signal neither multipliers. These actually are key advantages when compared with a lock-in amplifier and synchronous detectors for example.

**Figure 11.**
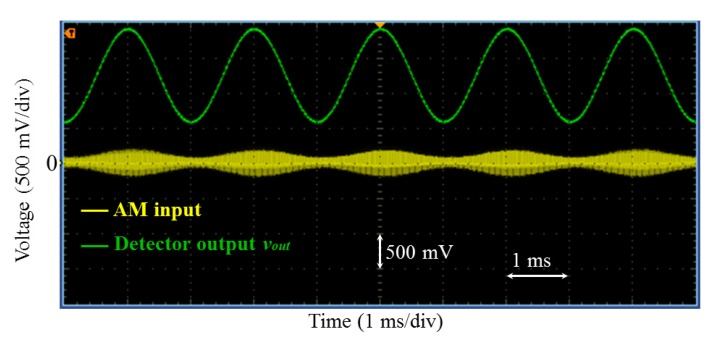
Demodulation of AM signals. The frequencies of the carrier and of the modulating signals were about 1 MHz and 400 Hz, respectively.

Furthermore, amplitude-modulated (AM) signals were also successfully demodulated as it is illustrated in example of Figure 11 where the frequencies of the carrier and the modulating signals were about 1 MHz and 400 Hz, respectively.

The −3 dB demodulation bandwidth in open-loop is mainly defined by the RC low-pass filter and by the gain-bandwidth product of inverter amplifier *U_2_*. For the values of the parameters given in Figure 6 this measured bandwidth was about 5 kHz.

In closed-loop, this bandwidth is expected to be enlarged. For a carrier frequency of 1 MHz and for *R_G_* = 10 kΩ, the expected bandwidth at −3 dB may be more than 50 kHz. More investigations and a comprehensive study of this aspect are necessary in order to proceed well beyond these first measurements and to establish the exact mathematical relationship between the demodulation bandwidth in open- and closed-loops and their dependence on the gain of the detector. This study, which is the subject of our current work, must consider the frequency dependence of transfer function, ***T***, in Equation (3) and the exact expression of transfer function of the closed loop in Equation (2). In any case, the demodulation bandwidth is larger than the one that could be realized by the RMS-to-DC converter, and it should be at least comparable to the bandwidth that could be achieved by other classical demodulators.

## 3. Application of the Amplitude Detector in the GMI Sensor

The developed detector was successfully integrated and used in a GMI sensor which was basically dedicated for electrical current measurements by GMI (Figure 1).

**Figure 12.**
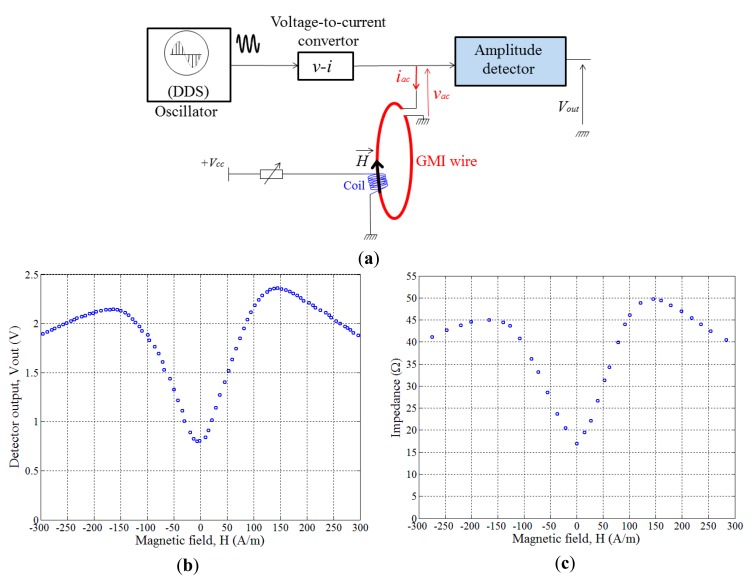
(**a**) Experimental setup; (**b**) The detector output, *V_out_*, as a function of the applied magnetic field; (**c**) The GMI curve measured using an impedance analyzer. The absolute value of the gain of the detector, 1/*G*, was fixed to about 10.

The GMI response of the GMI wire has been obtained in terms of voltage using the developed detector. For doing so, an external magnetic field *H⃗* has been created by a coil wound around the whole core as it is shown in experimental setup in Figure 12a (where only a schematic of the wound coil in illustrated). The length of this wound coil is slightly larger than the length of the wire to ensure the application of as homogenous circumferential field as possible in each point of the GMI wire. This is mandatory; otherwise, some portions of the wire would be in different magnetic states and it will be difficult to understand the behavior of the GMI wire.

The detector output, *V_out_*, was recorded as a function of this field (Figure 12b). In these experiments, a high frequency current, *i_ac_*, of 5 mA amplitude and 1 MHz frequency was used. The gain of the amplitude detector was fixed to about 10. This same GMI curve was obtained using an impedance analyzer as it is shown in Figure 12c. It is straightforward to verify that the value of the detector output is no more than the product of the impedance with the detector gain and the high frequency current *i_ac_*. For example, GMI curve in Figure 12b exhibits a minimum value of about 0.8 V at zero field and 2.4 V at a field of 145 A/m. This yields impedances of about 17 Ω and 49.4 Ω, respectively (Figure 12c).

These results show that the developed detector ensures a reliable measurement of the impedance. Both curves of Figure 12b,c exhibit the same aspect; even the slight intrinsic asymmetry is retained. This asymmetry in the GMI response has made the subject of a large number of publications and it could have various origins [1]. In our case, the main origin of this small asymmetry is attributed to the hysteresis of the magnetic materials (asymmetry with respect to the increasing and decreasing magnetic field) [1]. The curve in Figure 12b,c were obtained for an increasing magnetic field. For decreasing magnetic field, the asymmetry is reversed.

These GMI curves can also be obtained for other excitation frequencies knowing that the −3 dB high frequency bandwidth (with respect to the carrier) of the detector is measured at more than 20 MHz. In our experiments, the frequency of 1 MHz is used as an example of illustration only. This does not change the generality of the concept.

**Figure 13.**
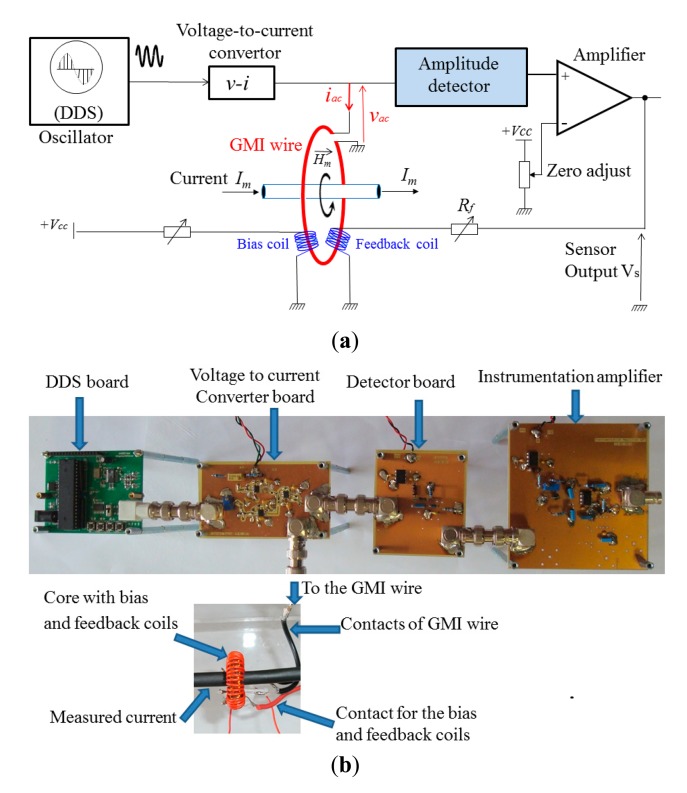
Block diagram of the GMI sensor for the measurement of magnetic fields (**a**) and photo of the setup (**b**). The GMI wire was biased with a magnetic field of about 55 A/m. A 100-turns feedback coil was used. The current *I_m_*, which produces the measured field, crosses the core.

Furthermore, the developed sensor was used to measure magnetic fields (produced by electrical currents) in open- and closed-loop of the GMI sensor as illustrated in Figure 13.

The GMI wire was biased at about 55 A/m by using a bias coil wound around the whole core. In this bias point, the sensitivity was about 0.0164 V/A/m.

The detector output was applied to the input of the instrumentation amplifier (AD620) that ensures more amplification and setting the output at zero volt for a zero measured magnetic field.

In closed-loop configuration, the output voltage of this amplifier is converted into a current through the feedback resistor, *R_f_*. This current is used to generate the feedback magnetic field thanks to a 100-turns feedback coil which was wound around the whole core. The current *I_m_*, that produces the measured field, crosses the core.

**Figure 14.**
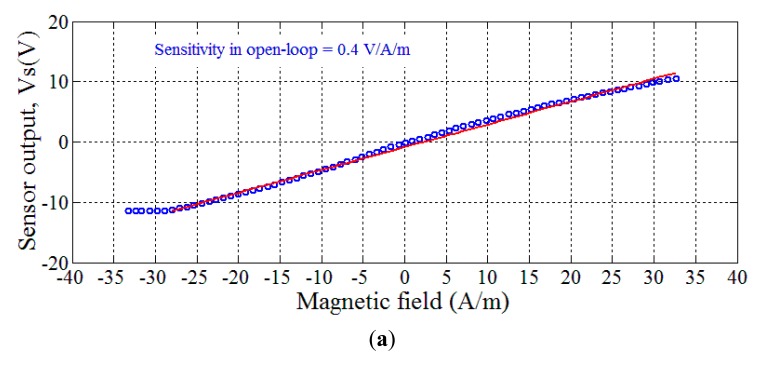

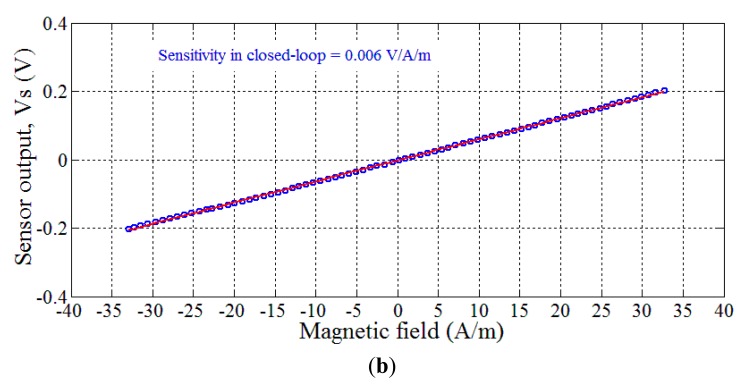
Sensor output as a function of the magnetic field (produced by the current *I_m_*) using the GMI sensor in (**a**) open-loop and (**b**) closed-loop. The red lines are linear fittings of the data.

Figure 14a shows the open-loop sensor output, *V_s_*, as a function of the DC measured field. The sensitivity in open-loop was about 0.4 V/A/m. This yields a sensitivity of 6.4 V/A when expressed in terms of the measured current *I_m_*. The linearity error obviously depends on the considered dynamic range. This error can be quantified by realizing a linear fitting (red lines in figure) between the sensor output and the measured field. The correlation coefficient α^2^ is then calculated. The linearity error is defined by the Equation (8):

(8) $$\text{linearity error}(\%)=\left(1-\alpha^{2}\right)\times 100\left(\%\right)$$

A perfect linearity is obtained when α^2^ = 1. In the case of Figure 14a, the correlation coefficient was about 0.9974. This yields a linearity error of 0.5% relative to a full scale (FS) of ±30 A/m.

The sensor output was also measured in closed-loop configuration with a feedback resistor *R_f_* = 10 Ω. The results are shown in Figure 14b. It is clear that the linearity of the sensor is largely improved in the considered dynamic range of ±33 A/m when compared with the open-loop operation. The correlation coefficient was about 0.9999 and the linearity error was of 0.02% FS.

**Figure 15.**
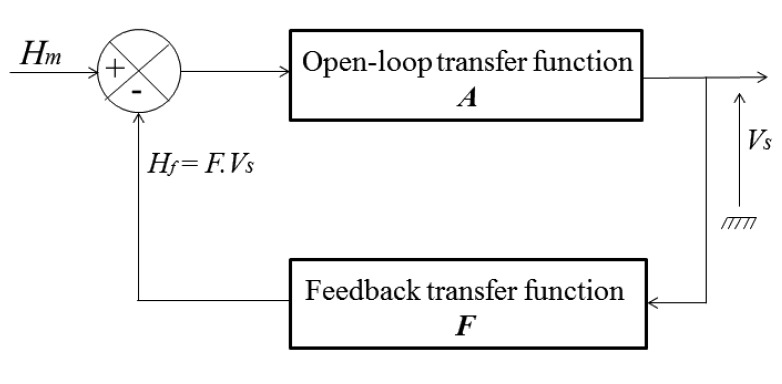
The functional diagram of the closed-loop of GMI sensor.

The sensitivity of the sensor is however naturally reduced in closed-loop. This measured sensitivity was about 0.006 V/A/m (or 0.1 V/A when expressed in terms of the measured current *I_m_*). It was in good agreement with the theoretical prediction. Actually, this sensitivity can be easily deduced from the functional block diagram of the sensor in closed-loop (Figure 15) [34,35].

In this diagram, ***A*** and ***F*** denote the transfer functions of the open-loop and of the feedback, respectively. *H_f_* is the feedback magnetic field resulting from the feedback current. In a similar way as in paragraph *2.2*, the transfer function of this closed-loop system is expressed by Equation (9):

(9) $$\frac{V_{s}}{H_{m}}=\frac{A}{1+F\cdot A}$$

When *A·F* ≫1, then V_s_/H_m_≈1/*F*, and the output depends linearly on the measured magnetic field *H_m_* with a proportionality constant of 1/*F*. Since this constant depends only on the parameters of the feedback, the linearity of the sensor is improved. Hysteresis and instabilities of the open-loop are also greatly reduced. In our design the transfer function of the feedback, ***F***, is given by (10):

(10) $$F=\frac{N}{\pi \cdot d\cdot R_{f}}$$

where *N* is the number of turns of the feedback coil.

With *N* = 100 turns, *d* = 2 cm and *R_f_* = 10 Ω, a simple calculation yields *F* = 160 A/m/V and A · F ≈ 24 ≫ 1. In this case, the sensitivity in close-loop is then 1/*F* = 0.0063 V/A/m, which is in very good agreement with the measured value.

Measurements of AC fields have also been conducted. The sensor demodulation bandwidth is obviously related to the detector bandwidth and to the frequency of the carrier signal. In open-loop of the GMI sensor, this bandwidth is close to the detector bandwidth (more than 50 kHz for a carrier frequency of 1 MHz). In closed-loop of the GMI sensor, the bandwidth is naturally increased by the quantity (1 + *F·A*) [34]. However, reliable measurements of this close-loop bandwidth require increasing the carrier frequency (the frequency of the carrier should generally be much larger than the frequency of the modulating signal).

It is also important to note that the paper was focused on the design of a new detector that can be used with a given and any GMI element. The optimization of the GMI element itself, while of great importance, was not the main scope of the current study. The GMI sensing head could be previously optimized to ensure higher intrinsic sensitivity and linearity in open-loop. Actually, the configuration of a ring shape in the current configuration may induce stresses in the amorphous wire which may decrease the intrinsic sensitivity and the linearity. The improvement of the ring magnetic properties could be performed using a stress-relief annealing techniques (electrical current annealing [36], tension current annealing…).

On the other hand, the sensitivity of a final GMI sensor also depends on a very large number of parameters such as the sensitive element composition and form (wire, ribbon, nanocrystalline), material treatment, dimensions, geometric configuration, the excitation conditions (*i.e.*, frequency and value of the *i_ac_* current, use or not of a DC bias current to reduce the magnetic noise), the way we detect the GMI voltage (on-diagonal or off-diagonal GMI), *etc.* When all these parameters are optimized, the GMI sensor using the developed detector will at least provide the same sensitivity and bandwidth as other detectors, but with the advantages of low complexity compared to a lock-in amplifier and possibility to measure very low signals with better linearity than the conventional diode-based detector.

It is still therefore necessary to proceed well beyond the first obtained results presented in the manuscript in order to take into account all these considerations. The accurate quantification of the contribution of this detection system in the final signal-to-noise ratio (SNR) and the resolution of the GMI sensors must also be performed. Since the detector is able to measure very weak signals, other related works in this area may concern the combination of the detector with preliminary carrier reduction techniques before rectification. This should enhance the detection sensitivity of the GMI sensors.

One can finally note that in embedded sensor systems applications, the power consumption of the sensor could also be an issue. In the current design, except for the DDS and its voltage-to-current converter, the whole system power consumption of the electronics was evaluated at a few tens of mW, which is close to the general power consumption of GMI sensors reported in the literature [1]. The use of the DDS, that requires also the use of a microcontroller, increases the total power consumption up to about several 100 mW. However, the use of DDS is not mandatory and it will not be the adequate solution if power consumption is an issue. In such a case, other classical oscillators and a simple voltage-to-current converter (using an injection resistor) can be used to maintain low power consumption. In other words, the new amplitude detector would not substantially increase the power consumption of GMI sensors beyond the current-state-of-the art.

## 4. Conclusions

We have presented an original concept of a high-frequency amplitude detector and demodulator with outstanding performance using a limiting op-amp. This detector did not make use of diodes or analog multipliers. It performed the measurement of low-level and high-frequency voltages. Both sine waveforms and pulsed signal have been successfully rectified. The use of the concept of feedback in this detector circuit allowed the input amplitude measurement with an easily adjustable gain. This detection gain depends only on passive components (resistor). The experimental test results were close to those of the developed theoretical model of the detector. The detector was successfully integrated in a GMI sensor prototype. Magnetic field measurements in both open- and closed-loop have been performed. Further works should address a comprehensive study of the detector and sensor bandwidth as well as a quantification of the detector performance in terms of the SNR. Other work should also focus on the optimization of the magnetic proprieties of the wire in ring configuration using annealing techniques.
